# The transcriptome variations of *Panaxnotoginseng* roots treated with different forms of nitrogen fertilizers

**DOI:** 10.1186/s12864-019-6340-7

**Published:** 2019-12-24

**Authors:** Xiaohong Ou, Shipeng Li, Peiran Liao, Xiuming Cui, Binglian Zheng, Ye Yang, Dahui Liu, Yun Zheng

**Affiliations:** 1 Kunming Key Laboratory of Sustainable Development and Utilization of Famous-Region Drug, Key Laboratory of Panax notoginseng Resources Sustainable Development and Utilization of State Administration of Traditional Chinese Medicine, Faculty of Life Science and Technology, Kunming University of Science and Technology, Kunming, 650500 China; 2 Yunnan Key Lab of Primate Biomedicine Research; Institute of Primate Translational Medicine, Kunming University of Science and Technology, Kunming University of Science and Technology, Kunming, 650500 China; 3 Guizhou University of Traditional Chinese Medicine, Guiyang, 550025 China; 4 College of Pharmacy, Hubei University of Chinese Medicine, Wuhan, 430065 China; 5 State Key Laboratory of Genetic Engineering, Collaborative Innovation Center for Genetics and Development, Institute of Plant Biology, School of Life Sciences, Fudan University, Shanghai, 200438 China

**Keywords:** *Panax notoginseng*, Transcriptome, Nitrate, *ACLA-3*, Ammonium stress

## Abstract

**Background:**

The sensitivity of plants to ammonia is a worldwide problem that limits crop production. Excessive use of ammonium as the sole nitrogen source results in morphological and physiological disorders, and retarded plant growth.

**Results:**

In this study we found that the root growth of *Panax notoginseng* was inhibited when only adding ammonium nitrogen fertilizer, but the supplement of nitrate fertilizer recovered the integrity, activity and growth of root. Twelve RNA-seq profiles in four sample groups were produced and analyzed to identify deregulated genes in samples with different treatments. In comparisons to NH${~}_{4}^{+}$ treated samples, *ACLA-3* gene is up-regulated in samples treated with NO${~}_{3}^{-}$ and with both NH$_{4}^{+}$ and NO${~}_{3}^{-}$, which is further validated by qRT-PCR in another set of samples. Subsequently, we show that the some key metabolites in the TCA cycle are also significantly enhanced when introducing NO${~}_{3}^{-}$. These potentially enhance the integrity and recover the growth of *Panax notoginseng* roots.

**Conclusion:**

These results suggest that the activated TCA cycle, as demonstrated by up-regulation of *ACLA-3* and several key metabolites in this cycle, contributes to the increased *Panax notoginseng* root yield when applying both ammonium and nitrate fertilizer.

## Background

*Panax notoginseng* (Burk.) F. H. Chen (*P. notoginseng*) belongs to the *Araliaceae* genus and has been in domesticated cultivation for more than 400 years. *P. notoginseng* is a famous traditional Chinese medicine for treating cardiovascular and cerebrovascular diseases [1]. According to the statistics of 2016, *P. notoginseng* has been planted on approximately 53,000 hectares, from which around 50,000 tons of medicinal materials have been harvested, with an agricultural value of up to 1.5 billion dollars. The income from cultivation and processing, as well as from other relevant industries, has become one of the most important economic pillars of Yunnan province. However, since the high values of *P. notoginseng*, abusing nitrogen (N) fertilizers to harvest more raw material is very common [2, 3]. Abusing N fertilizers has brought some problems, such as imbalanced of soil nutrients and microbe [3], which could aggravate continuous cropping obstacle [4]. Many studies have been carried out on continuous cropping obstacle till now [5–7]. Furthermore, the average N application rate in *P. notoginseng* cultivation is approximately 250 kg ∗hm^−2^ [8], causes soil available N with an average of 213.65 mg ∗kg^−^^1^ [9], which is 42.4% higher than the standard of extremely rich level ($\geqslant $150 mg ∗kg^−1^). Besides, our previous survey shows abusing N fertilizers causes NH _4_^+^ concentration of some cultivation soils reaches up to 6 mM, which would increase the incidence rate of ammonium toxicity (unpublished data).

Ammonium toxicity is a common phenomenon in agricultural system, and there are a few measures to prevent or alleviate it [10–12]. Among those measures, ammonium and nitrate combined application is thought to be an effective way to prevent ammonium toxicity, but its mechanism is not unified yet [13]. For instance, Roosta et al. [14] suggested that nitrate addition can increase cytokinin synthesis and translocation to alleviate ammonium toxicity of cucumber plants. However, Yang et al. [15] indicated that *A. thaliana* with ammonium and nitrate combined application has a higher auxin content, rather than cytokinin compared to that with ammonium alone. Moreover, nitrate-dependent alleviation of ammonium toxicity in *A. thaliana* is linked to nitrate signaling, uptake and reduction [16], anion channel *SLAH3* [13].

With the development of RNA-seq technology, some valuable information can be gained easily by using this technology. Such as, with RNA-seq analysis, Yang et al. [17] have indicated that rice treated with 10 mM ammonium has 63 differentially expressed genes (DEGs) in root and 115 DEGs in leaf compared to that with no ammonium; Sun et al. [18] have reported that rice with 7.5 mM ammonium treated for 4 h would generate 307 DEGs, involve in carbohydrate, photosynthesis, secondary metabolism, etc., by which the ammonium tolerance of rice can be improved. Moreover, Yang et al. [19] have used RNA-seq to compare the different DEGs of rice with combined treating of ammonium and nitrate and with no N treating. Similarly, analysis the different DEGs of plants treated with ammonium alone and that with combination of ammonium and nitrate, would get some useful information on nitrate-dependent alleviation of ammonium toxicity, but few reports have reported that till now.

Therefore, based on the ammonium toxicity in *P. notoginseng* cultivation, the inhibitory of growth phenotypes with NO3− addition were studied by a sandy or hydroponic culture. It showed that the combined application of ammonium and nitrate can alleviate ammonium toxicity of *P. notoginseng*. After that, we identifies thousands of DEGs genes under treatments of different nitrogen forms using RNA-seq. Among up-regulated genes in both nitrate only and nitrate plus ammonium treatments, *ACLA-3*, ATP-citrate lyase A-3, and a few other genes were validated to be up-regulated by qRT-PCR experiments. *ACLA-3* is the primary enzyme responsible for the synthesis of cytosolic acetyl-CoA in the TCA cycle. Because of the up-regulation of *ACLA-3*, we examined several metabolites in the TCA cycle, which are also up-regulated in both nitrate only and nitrate plus ammonium treatments. These findings are consistent with the activation of TCA cycle when supplying nitrate in other plant species [20]. These results would guide N fertilization in *P. notoginseng* cultivation, contributes to the recovery of *P. notoginseng* root growth when supplying nitrate to *P. notoginseng* root under ammonium stress.

## Results

### Nitrate alleviates the inhibition on root growth of *P. notoginseng* under ammonium toxicity

At first, we observed the ammonium toxicity phenomenon in a field survey, and then in a sandy culture. To explain the mechanism of the toxicity and to speed up the search procedure, sandy culture and hydroponic culture were used. From the results, we noticed that the phenotypes of the sandy and hydroponic experiments were the same, so we used sandy culture for the experiments to examine physiological status and used hydroponic culture for experiments to examine hair root growth when the plants were treated with different forms of nitrogen fertilizers.

Growth of seedlings with sandy culture was showed in supplementary file (see Additional file 2: Figure S1). We observed that the leaf of CK (without N) was a little chlorosis in the leaf margin, which was caused by N deficiency. However, leaf of the 15A treatment (with 15 mM NH 4+) was totally chlorosis, which was a typical symptom of ammonium toxicity. Both the 15N treatment (with 15 mM NO _3_^−^) and the 15AN treatment (with 15 mM NH _4_^+^+15 mM NO _3_^−^) had a green leaf, indicating that seedlings with these two treatments had neither N deficiency nor ammonium toxicity. The plant height, hair root length, rhizome diameter, root diameter, shoot and root biomass of seedlings were significantly inhibited under 15A treatment comparing with those measures in CK (see details in Additional file 2: Figure S2 and Additional file 1: Table S1). Furthermore, the 15N treatment had significant promotion effects on the plant height, hair root length, root diameter, shoot and root biomass of seedlings (see details in Additional file 2: Figure S2 and Additional file 1: Table S1). The 15AN treatment also significantly increased the plant height, hair root length, root biomass of seedlings (see details in Additional file 2: Figure S2). In summary, nitrate recovers the growth of *P. notoginseng* seedlings under NH${~}_{4}^{+}$ stress.

**Fig. 1:**
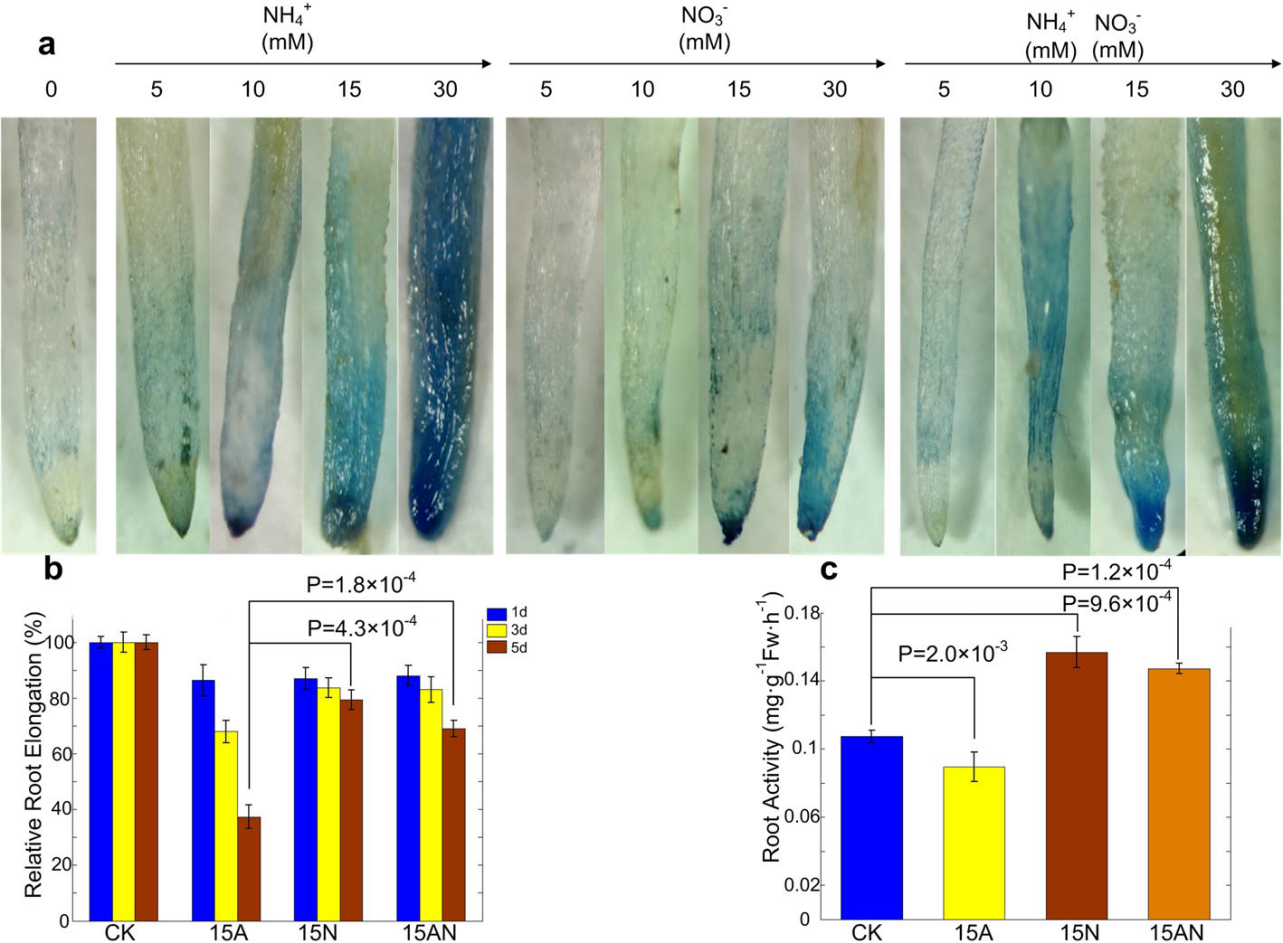
Effects of different forms of nitrogen treatments with different concentration gradients on root tips of *P. notoginseng*. **a** The plasma membrane integrity of root tips of *P. notoginseng* under different treatment conditions, Numbers represent different concentration gradients. **b** The Relative Root Elongation of root tips of *P. notoginseng* under different forms of nitrogen treatments. The 1d, 3d, and 5d represent the first day, the third day, and the fifth day, respectively. **c** The Root Activity of *P. notoginseng* Effect under different forms of nitrogen treatments

A hydroponic culture was conducted to test the effects of different nitrogen forms on hair root growth of *P. notoginseng* seedlings (Fig. 1). E-B staining analysis of root tips showed that plasma membrane of root tip cells was gradually damaged with the nitrogen concentration increasing, because the degree of blue color represents the degree of root damage. The damage degree caused by ammonium treatment was the most serious, the second one was the sample treated with ammonium combined nitrate, and minor effects were observed under nitrate treatments (Fig. 1a). The relative root elongation (RRE) under 15A treatment was approximately 37%, which was significantly lower than that under 15N and 15AN treatments (*P*=4.3×10^−4^ and *P*=1.8×10^−4^, respectively, Student’s *t*-test), respectively, after five days (Fig. 1b and Additional file 1: Table S2). Meanwhile, the root activity of 15A treatment was significantly lower than that of CK (*P*=2.0×10^−3^, Student’s *t*-test), and significantly lower than those under the 15N and 15AN treatments (*P*=2.5×10^−4^ and *P*=2.7×10^−5^, respectively, Student’s *t*-test), respectively (Fig. 1c and Additional file 1: Table S3). All results above indicated that *P. notoginseng* seedlings treated with ammonium had a visible toxicity effects, i.e., root tip damage, root elongation and growth inhibition, as well as low biomass, and nitrate could alleviate the toxicity symptom caused by ammonium.

### RNA-Seq profiles of *P. notoginseng* roots under different forms of nitrogen treatments

According to results of E-B staining analysis for plasma membrane of *P. notoginseng* root tips, we hypothesized that 15A, 15N, and 15AN treatments could significantly affect the metabolism of *P. notoginseng*, without producing significant damage. Therefore, transcriptome analysis of *P. notoginseng* roots were performed under the treatments with 15 mM for each condition (Fig. 1a).

We generated 12 RNA-seq libraries from the pooled RNA samples isolated from *P. notoginseng* roots, with 3 samples for CK, 15A, 15N, and 15AN, respectively. Plants were grown under greenhouse conditions without special treatments. These 12 RNA-seq libraries were sequenced using Illumina HiSeq 2000 sequencer, and approximately 20 million reads were obtained for each of the libraries. After examining the scores per nucleotides with FASTQC, the qualities of the obtained RNA-seq sequencing profiles were generally good with scores (see Additional file 2: Figure S3).

### *P. notoginseng* roots under the same treatments have similar gene expression profiles

The obtained sequencing data were aligned to the *P. notoginseng* genome [21] and assembled with the Cufflinks pipeline [22] (see details in Materials and methods). We obtained approximately 95% mapped rate to the reference genome [21] (see details in Additional file 1: Table S4).

We totally obtained 146,330 assembled genes of which 138,976 overlapped to annotated genes reported previously [21] (Additional file 2: Figure S4a) and 7354 were novel and did not overlap to previously annotated genes (Additional file 2: Figure S4a), suggesting the annotation of the reported genome is incomplete. Our assembly of transcript did not cover 1764 genes reported previously. The average abundances of the assembled genes were between 10 to 100 FPKM (Additional file 2: Figure S4b). The average expression levels of most genes are smaller than 500 FPKM in all four experimental groups (Additional file 2: Figure S4c to S4f).

**Fig. 2:**
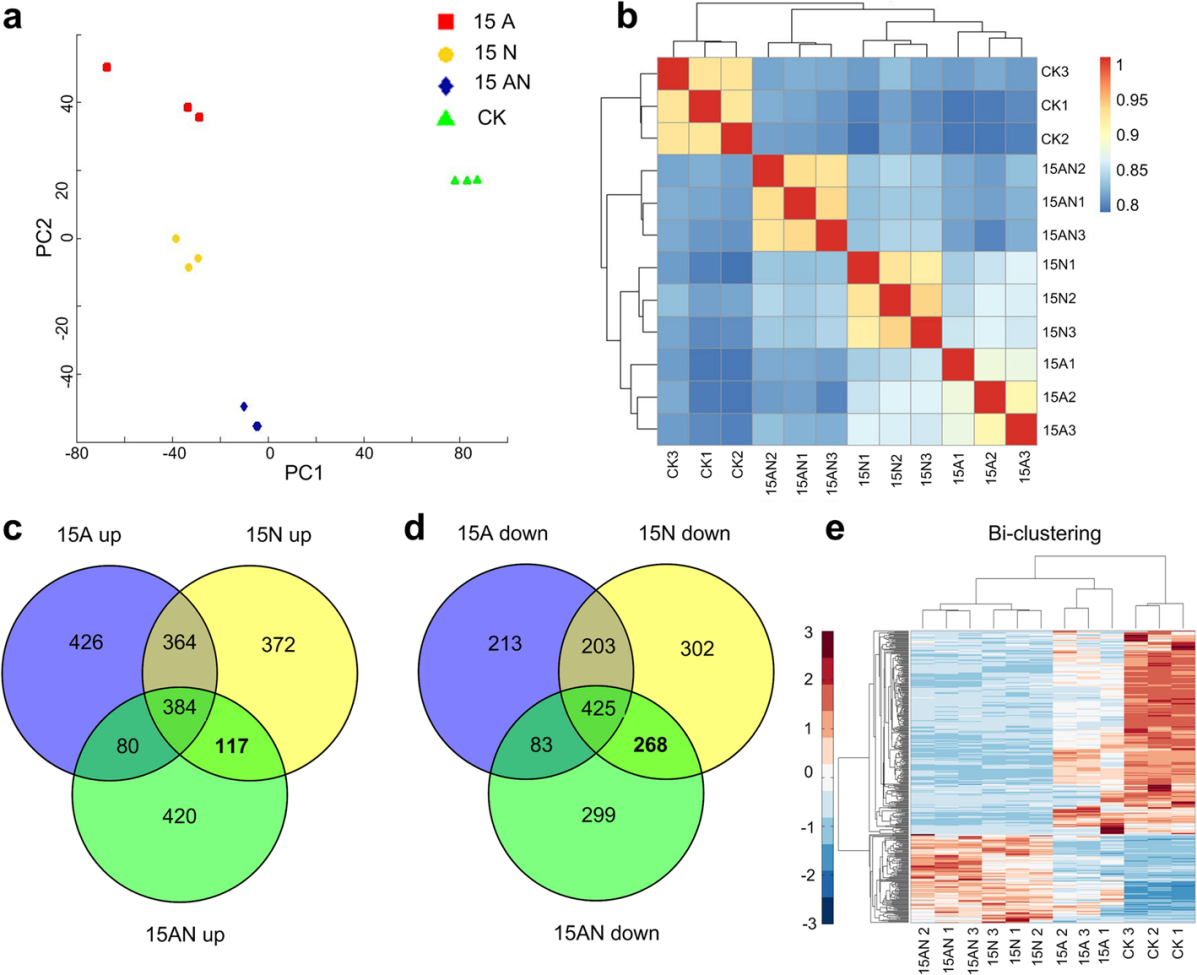
Differentially expressed genes in different forms of nitrogen treatments in *P. notoginseng*. **a** The PCA analysis of RNA-Seq expression profiles in different treatment conditions. **b** The hierarchical clustering of RNA-Seq expression profiles in different conditions. **c** and **d** The number of up-regulated genes and down-regulated identified under different conditions in this study, respectively. **e** The up-regulated and down-regulated genes bi-clustering of RNA-Seq expression profiles in different treatment conditions

The genes with average abundances and variances of abundances of at least 5 FPKM (Additional file 1: Table S5) were selected to perform Principle Component Analysis (PCA) and Hierarchical Clustering. The samples from the same treatments were clustered together and samples from different treatments were clearly differentiated (Fig. 2a). We also found the strong correlation among the samples of the same treatment, meanwhile samples from different processing conditions have much lower correlation coefficient values (Fig. 2b). These results suggest that samples in the sample groups have similar gene expression patterns and a good repeatability of the RNA-seq profiles.

### Differentially expressed genes in different forms of nitrogen treatments in *P. notoginseng* roots

In order to explore the mechanisms by which the toxicity of ammonium was reduced after nitrate treatments, we identified differentially expressed genes (DEGs) in different treatments when compared to control using the edgeR package [23] (see details in Material and methods). The significantly differently expressed genes were identified with False Discovery Rate (FDR) values of <0.05 and absolute log-scaled fold change (|log_2_FC |) >1 (Additional file 1: Table S6 to S11). The numbers of DEGs were shown in Fig. 2c and d.

According to the results of physiological experiments, we noticed that nitrate could alleviate ammonium toxicity. Thus, we selected 117 up-regulated and 268 down-regulated genes under 15N and 15AN treatments but not under 15A treatment to perform a Biclustering analysis (Fig. 2e). The results show that the sample of different groups were correctly clustered, suggesting these genes contribute to the differences in different groups.

### Functional analysis of differentially expressed genes

DEGs in different treatments were used to identify enriched Gene Ontology (GO) terms (Additional file 1: Table S12 to Table S17) and KEGG pathways (Additional file 1: Table S20 to Table S25). Similarly, the number of enriched GO terms and KEGG pathways enriched in DEGs were shown under different treatments (see details in Additional file 2: Figure S5 and Materials and methods).

The common GO terms in up-regulated DEGs in 15N and 15AN but not in 15A include vacuolar membrane (GO:0005774), vacuolar part (GO:0044437), cofactor metabolic process (GO:0051186), cellular response to water deprivation (GO:0042631), cellular response to abiotic stimulus (GO:0071214) and so on (Additional file 1: Table S18 and Additional file 2: Figure S5a).

Similarly, the common GO terms of the down-regulated DEGs in 15N and 15AN but not in 15A include photosynthesis, light reaction (GO:0019684), photosynthesis, light harvesting in photosystem I (GO:0009768), generation of precursor metabolites and energy (GO:0006091), carbohydrate metabolic process (GO:0005975), sucrose metabolic process (GO:0005985), amino acid catabolic processes (GO:0009065, GO:0009068, GO:1901606), nitrogen transport (GO:0015706), and carbon metabolism processes (GO:0015977, GO:0005975). Among the cellular components category, significant GO terms include photosystem I (GO:0009522), chloroplast thylakoid membrane plastid (GO:0009535), thylakoid membrane (GO:0055035), and so on. For the molecular function, significant GO terms include pigment binding (GO:0031409) and nitrate transmembrane transporter activity (GO:0015112) which was involved in nitrogen metabolism (Additional file 1: Table S19 and Additional file 2: Figure S5c).

Meanwhile, the common KEGG pathways in 15N and 15AN but not in 15A are TCA cycle (ath00020), Plant hormone signal transduction (ath04075), and Taurine and hypotaurine metabolism (ath00430) for up-regulated genes (see details in Table 1 and Additional file 2: Figure S5b). In down-regulated genes, the common KEGG pathways in 15N and 15AN but not in 15A are Photosynthesis (ath00195), Glutathione metabolism (ath00480), Amino sugar and nucleotide sugar metabolism (ath00520), Starch and sucrose metabolism (ath00500), and Porphyrin and chlorophyll metabolism (ath00860) (see details in Table 1 and Additional file 2: Figure S5d).

**Table 1:** KEGG pathways of common DEGs between 15N and 15AN

	KEGG pathway	ID	15N			15AN		
			Input	Total	*P*-Value	Input	Total	*P*-Value
Up-regulated DEGs	Citrate cycle (TCA cycle)	ath00020	6	63	1.18E-2	7	63	5.32E-4
	Taurine and hypotaurine metabolism	ath00430	3	14	1.06E-2	3	14	4.53E-3
	Plant hormone signal transduction	ath04075	17	271	2.79E-3	12	271	1.32E-2
Down-regulated DEGs	Photosynthesis	ath00195	12	77	1.26E-5	10	77	9.74E-5
	Glutathione metabolism	ath00480	8	93	1.04E-2	7	93	1.66E-2
	Starch and sucrose metabolism	ath00500	13	202	1.24E-2	11	202	2.63E-2
	Amino sugar and nucleotide sugar metabolism	ath00520	10	135	1.15E-2	9	135	1.43E-2
	Porphyrin and chlorophyll metabolism	ath00860	5	48	2.02E-2	4	48	4.78E-2

**Fig. 3:**
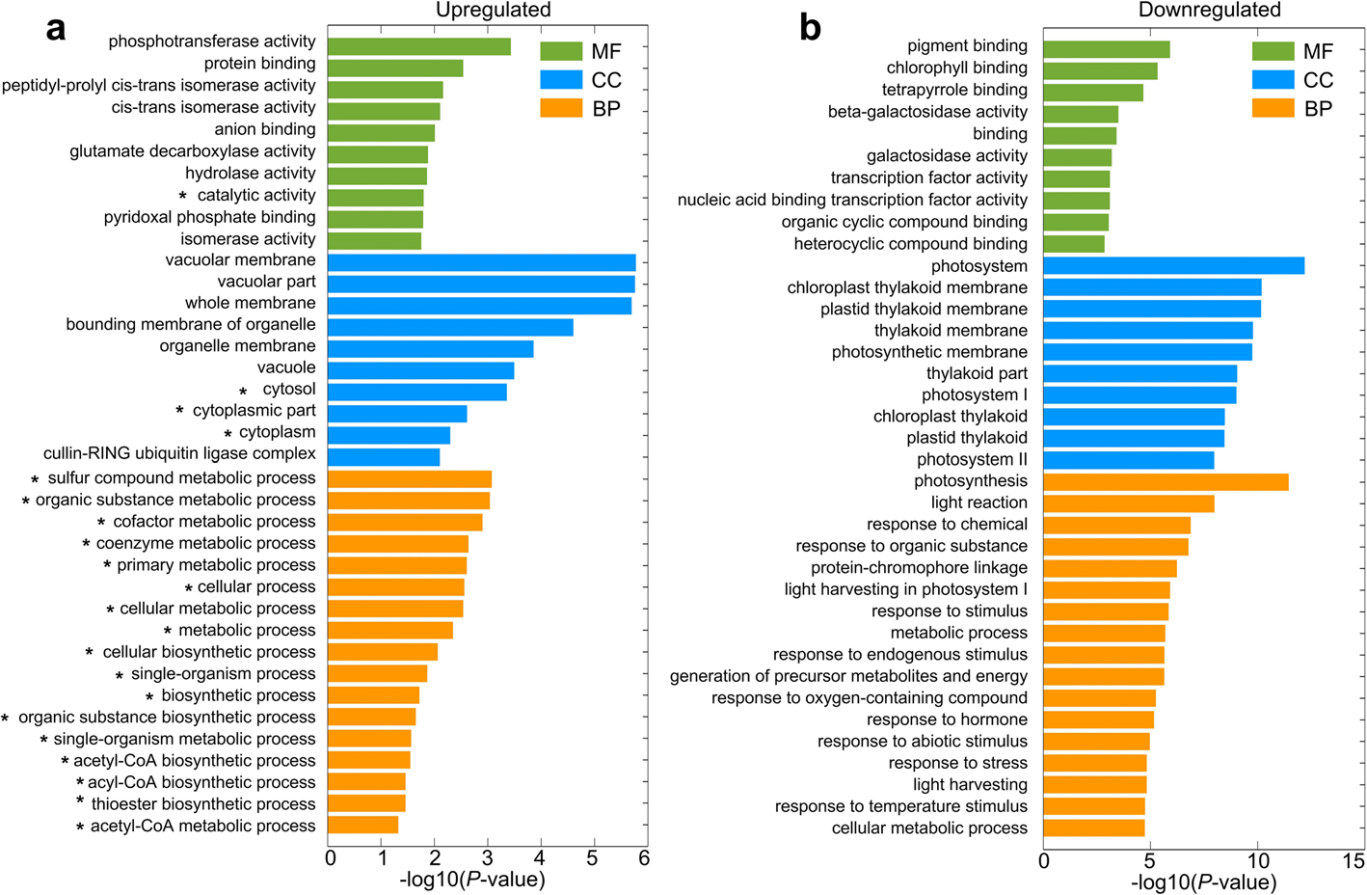
Enriched GO terms for the commonly deregulated genes in 15N and 15AN treatments but not in 15A treatment. **a** The enriched GO terms of 117 up-regulated genes. The asterisk represents the pathway in which *ACLA-3* is involved. **b** The enriched GO terms of 268 down-regulated genes

Because the supplements of nitrate alleviate the ammonium toxicity, we examined the 117 and 268 up- and down-regulated genes that are shared in the 15N and 15AN treated groups and not in 15A treated group, and analyzed the enriched GO and KEGG pathways of these genes (as shown in Fig. 3). *ACLA-3* is one of the commonly up-regulated genes and appears in many significant common GO terms and KEGG pathways of 15N and 15AN treated groups (see Fig. 3a). Therefore, we choose to further validate its expression. Furthermore, the common up-regulated genes of 15N and 15AN treated groups are enriched in the TCA cycle (Additional file 1: Table S26), in which *ACLA-3* is also involved.

As shown Fig. 3b, the 268 down-regulated genes are enriched in many GO terms related to photosynthesis, which was consistent with the common GO terms in 15N and 15AN but not in 15A (Additional file 1: Table S19).

### Validation of the expression of DEGs that may contribute to the alleviated ammonium toxicity

**Fig. 4:**
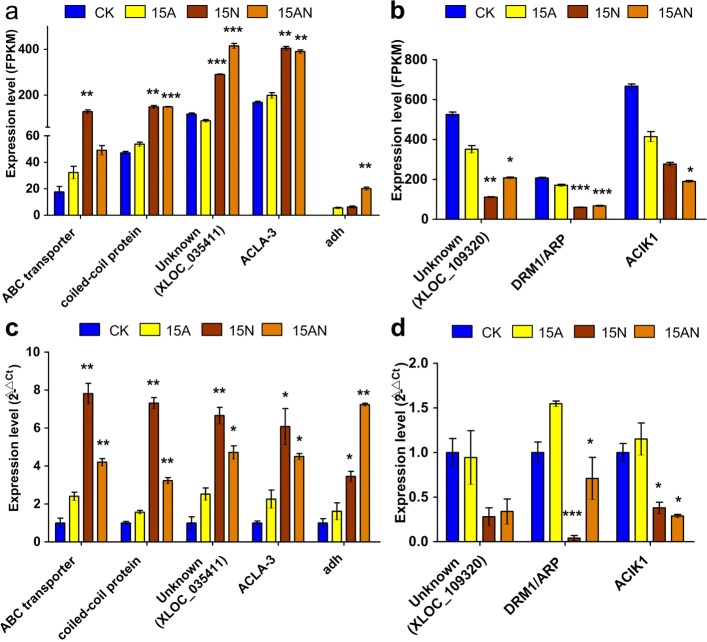
Validating expression patterns of up- or down-regulated genes in the 15N and 15AN treatments but not in 15A treatment by qRT-PCR in *P. notoginseng*. All values shown are mean values ± standard errors (S.E.). The asterisks indicate significant differences compared with the 15A treatment under the same treatment conditions (Student’s *t*-test). Standard deviations were calculated from three technical replicates. The results shown were reproduced with three biological replicates. * represents *P*-values <0.05; ** represents *P*-values <0.01; *** represents *P*-values <0.001. **a** and **b** The up- and down-regulated genes in the 15N and 15AN treatments detected using RNA-seq profiles in *P. notoginseng*. (**c**) and (**d**) The expression levels of the up- and down-regulated genes in Part (**a**) and (**b**), respectively, verified using qRT-PCR

To verify the expression of several key DEGs that may contribute to alleviated ammonium toxicity, we performed quantitative RT-PCR (qRT-PCR) assays with independent samples collected from the *P. notoginseng* roots under different treatments (CK, 15A, 15N, and 15AN). We selected 10 genes that are commonly deregulated in 15N and 15AN treatments but not in 15A, i.e., 6 up-regulated and 4 down-regulated ones, identified in the RNA-Seq data (Fig. 4a and b, Additional file 2: Figure S6a to S6d, Additional file 1: Table S29 and Table S32), which potentially alleviate ammonium toxicity.

The qRT-PCR results show that four of the six commonly deregulated genes in 15N and 15AN, i.e., an ABC transporter, a coiled-coil protein, an unknown gene (XLOC_035411), and *ACLA-3*, show up-regulated expression levels in 15N and 15AN when compared to 15A using qRT-PCR experiments (Fig. 4c and Additional file 1: Table S29), which is the same as those in RNA-seq profiles. ADH, commonly up-regulated in 15A, 15N and 15AN treatments, also has up-regulated expression levels in 15N and 15AN in the qRT-PCR experiments. The expression of three of the four down-regulated genes, i.e., an unknown gene (XLOC_109320), DRM1/ARP, and ACIK1, in 15N and 15AN treatments were validated using qRT-PCR (Fig. 4d, Additional file 2: Figure S6d, and Additional file 1: Table S29). It should be noticed that the *P. notoginseng* plants used in qRT-PCR experiments were not the same as those used in RNA-seq profiling. Therefore there could be small discrepancy between the expression levels obtained in qRT-PCR experiments and those in RNA-seq profiles, such as those of ADH.

We also selected 9 and 6 genes that are commonly up- and down-regulated in 15A, 15N and 15AN treatments for verification using qRT-PCR (Additional file 1: Table S32). The qRT-PCR results show that the expression levels of these genes have no unified patterns (Additional file 2: Figure S6). After performing GO and KEGG pathway analysis for the 384 and 425 commonly up- and down-regulated genes in 15A, 15N and 15AN treatments, we found that the up-regulated genes are mainly enriched in various metabolic processes, including oxidoreductase activity, acting on paired donors, with oxidation of a pair of donors resulting in the reduction of molecular oxygen to two molecules of water (GO:0016717); unsaturated fatty acid biosynthetic process (GO:0006636); and unsaturated fatty acid metabolic process (GO:0033559). The enriched KEGG pathways of up-regulated genes include Biosynthesis of unsaturated fatty acids (ath01040) and Fatty acid metabolism (ath01212). And down-regulated genes are mainly involved in response to stresses, including response to stimulus (GO:0050896); response to chemical (GO:0042221); response to organic substance (GO:0010033). The enriched KEGG pathways of down-regulated genes include Metabolic pathways (ath01100) and Biosynthesis of secondary metabolites (ath01110). These results suggest that these genes probably contribute to the general stress responses and metabolic processes.

### Validation on metabolites involved in the alleviation pathway of *ACLA-3*

**Fig. 5:**
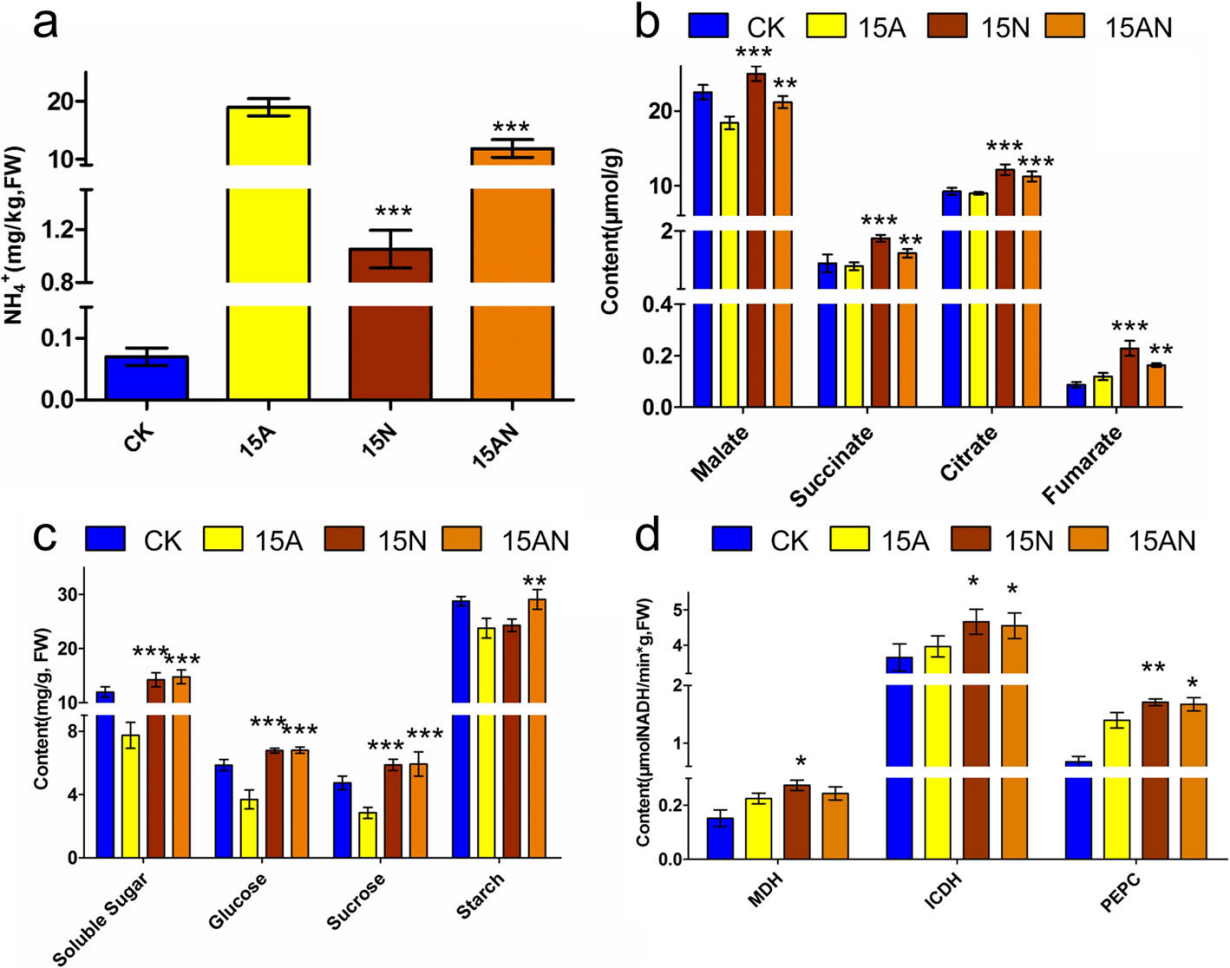
The contents of some metabolites associated with TCA metabolic pathway in *P. notoginseng* roots under different forms of nitrogen treatments. All values shown are mean values ± standard errors (S.E.). The asterisks indicate significant differences compared with the 15A treatment under the same treatment conditions (Student’s *t*-test). Standard deviations were calculated from three technical replicates. The results shown were reproduced with three biological replicates. * represents *P*-values <0.05; ** represents *P*-values <0.01; *** represents *P*-values <0.001. **a** Effects of the different forms of nitrogen treatments on changes of organic acids in TCA cycle of *P. notoginseng*. **b** The changes of organic acid synthases in TCA cycle. **c** The changes of major sugar compounds in TCA cycle. **d** The NH _4_^+^ content in TCA cycle

Previous studies have demonstrated an imbalance of carbon and nitrogen metabolism is an important mechanism for ammonium toxicity [24–26]. By using RNA-seq analysis, this study showed the gene, *ACLA-3*, played an important role in the TCA cycle, which could involve in the alleviation pathway of ammonium toxicity by regulating the carbon and nitrogen metabolism of *P. notoginseng*. Thus, this study determined the metabolites contents and enzymes activities, which might relate to the pathway involving the gene of *ACLA-3*. Compared to CK, the 15A treatment significantly increased the accumulation of free NH _4_^+^, which is mainly reason for imbalance carbon and nitrogen metabolism [24–26], however, the accumulation could be significantly decreased with the 15AN treatment (Fig. 5a). Moreover, compared to CK, the 15A treatment significantly decreased the content of organic acids (malate, citrate, succinate and fumarate, Fig. 5b) and carbohydrates (soluble sugar, glucose, sucrose and starch, Fig. 5c), as well as the activity of MDH, ICDH and PEPC of the TCA cycle (Fig. 5d), but all of those could be increased with the 15AN treatment (see more details in Additional file 1: Table S30 and S31, respectively). Among of the contents of main amino acid, Gln and Thr have decreased contents in 15N treatment when compared to 15A; Arg has a decreased content in 15AN treatment when compared to 15A (Additional 2: Figure S7). Thus, these results suggested that *ALCA-3* is involved in nitrate-dependent alleviation of ammonium toxicity by regulating related carbon metabolism.

## Discussion

### Nitrate alleviates the inhibitory effect of ammonium on the plant growth

Ammonium toxicity can inhibit the root growth, then restrain the development of aboveground, subsequently reduce crop production and may even result in death [10, 27]. Hoopen et al. [28] indicated that barley seedlings *(H. vulgare)* treated for 3 days with nutrition solution containing 1.5 mM NO _3_^−^, then transplanted to a complete nutrition solution containing 3 or 6 mM NH _4_^+^ for 5 days, the fresh root weight is decreased by 28%—32%, comparing with that treated with 1.5 mM NO _3_^−^. Li et al. [29] found that only the root tip or whole root of *A. thaliana* in contact with NH _4_^+^ containing medium can inhibit the root elongation. Liu et al. [30] found that the root tip cell length and division are evidently decreased in the meristem zone of *A. thaliana* when supplied with ammonium. Thus, the root specifically the root tip is the primary part that sensitive to ammonium toxicity. The present study suggested that *P. notoginseng* seedlings treated with ammonium alone can inhibit the growth and biomass accumulation obviously (Additional file 1: Table S1, S2, and S3, Fig. 1a, Additional file 2: Figure S1, S2). Furthermore, the root elongation and root activity of *P. notoginseng* seedlings are significantly inhibited by the increasing concentration of ammonium, and root plasma membrane also is severely damaged (Fig. 1b). However, addition of NO 3− can alleviate the inhibitory effect caused by ammonium (Fig. 1, Additional file 2: Figure S1, S2). These results are similar to that of Yang et al. [15], who found that *A. thaliana* seedlings treated with NH_4_NO_3_ as N source had an approximately 10-fold longer root length compared to that treated with NH 4+ as N source. Consequently, it is suggested that NO 3− can alleviate ammonium toxicity effects on the growth of *P. notoginseng* seedlings.

### The unbalanced metabolism of carbon and nitrogen caused by NH 4+ is one of the causes of poisoning to *P. notoginseng* roots

It is widely accepted that NH 4+ assimilated primarily into organic compounds via glutamine synthetase/glutamate synthase pathway (GS/GOGAT; GS, EC 6.3.1.2; GOGAT, EC 1.4.1.14) [31], which catalyzes NH 4+ and *α*-ketoglutarate to synthesize glutamate. In regular growing, the carbon/nitrogen metabolism of plant is in equilibrium. The NH 4+ absorbed by plants needs to be converted into amino acids by *α*-ketoglutarate as a carbon frame under the action of NH 4+ assimilation enzymes, and then converted into protein by plants.

However, the plant absorbs NH 4+, will consume a lot *α*-ketoglutarate. When the plant itself is low carbohydrate content, or when the amount of carbohydrates converted to *α*-ketoglutarate by the TCA cycle is insufficient for NH 4+ assimilation, it will cause an unbalance in the carbon and nitrogen metabolism of the plants, which in turn will cause poisoning to the plants. For example, Ariz et al. [26] study found that increased NH 4+ concentration results in reduced *Pisum sativum* Linn carbohydrate content as a cause of ammonium toxicity. Vega-Mas et al. [32] also considered that the cause of ammonium toxicity in tomato is that the large amount of NH 4+ assimilation leads to imbalance of its carbon and nitrogen metabolism. When exposed to ammonium stress, plants will initiate their own defense mechanisms to reduce the impact of ammonium stress on carbon and nitrogen metabolism imbalance. In that equilibrium, *α*-ketoglutarate in plant which is a intermediate product of TCA cycle, is enough for NH 4+ assimilation. Wherein, PEPC, MDH and ICDH are the key enzymes affecting the production of *α*-ketoglutarate during the TCA cycle [33, 34].

**Fig. 6:**
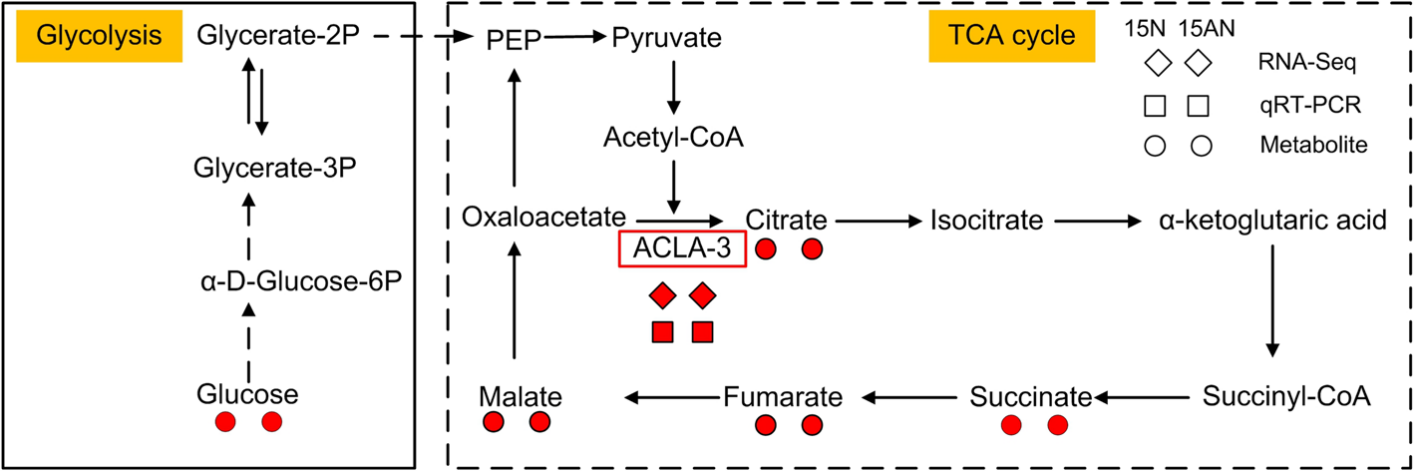
Schematic representation of glycolysis and TCA cycle in *P. notoginseng*. Solid arrows represent direct procedures. Dashed arrows represent indirect procedures. The red squares and red diamonds below the *ACLA-3* gene in the red rectangle mean that *ACLA-3* is up-regulated in 15N and 15AN treatments but not in 15A treatment as validated with qRT-PCR and RNA-seq, respectively. Red circles represent up-regulated metabolites

Interestingly, there is a widely consensus that elevating carbohydrate content can increase the organic acids synthetases activity of TCA cycle to alleviate ammonium toxicity. For instance, addition exogenous C can increase PEPC activity [35]; high irradiance improves carbohydrate content and organic acids synthetases activity [36]; elevated CO_2_ increases carbohydrate content and removes the inhibition of ammonium toxicity on organic acids synthetases activity [32, 37]. In addition, Setié et al. [38] indicated that tomato seedlings (Agora Hybrid F1) treated with 10 mM nitrate solution have higher *α*-ketoglutarate content than that treated with 10 mM ammonium solution. As shown above, our results indicate that NO 3− can up-regulate ACLA-3 and other compounds in the TCA cycle (Fig. 5a and 6), which subsequently increases the activity of organic acids synthetases (Fig. 5b), contents of carbohydrates (Fig. 5c). These potentially will increase the content of *α*-ketoglutarate for accelerating NH 4+ assimilation. Our results indicate that the content of NH 4+ was significantly decreased when adding nitrate, as shown in Fig. 5d.

### Nitrate alleviates ammonium toxicity by enhancing *ACLA-3*

The imbalance of carbon and nitrogen metabolism is an important mechanism for ammonium toxicity [26]. On the one hand, the imbalance is due to excessive free NH 4+ accumulation in plants, which needs massive carbohydrates to assimilated it [39, 40]. On the other hand, the imbalance is due to the reducing of carbohydrate accumulation, which is caused by inhibiting photosynthesis [41]. In view of this, decreasing the accumulation of NH 4+ or increasing the accumulation of carbohydrate is considered to be an effective way for ammonium toxicity alleviation. Studies have indicated that exogenous addition of CO 32− [14], HCO 3− [42], glucose [43] or increasing irradiance [36] or CO_2_ [32, 37] can improve the carbohydrate accumulation to alleviate ammonium toxicity by balancing carbon and nitrogen metabolism. Interestingly, all of these measures are related to the TCA cycle, which can provide *α*-ketoglutarate, a primary carbon skeleton for NH 4+ assimilation. For instance, Roosta et al. [14] have demonstrated addition CO 32− can improve the PEPC activity of cucumber seedlings under ammonium toxicity. Vega-Mas et al. [37] showed that increasing the ambient CO_2_ concentration also improves the synthase activity in the TCA cycle of tomato plants under ammonium stress. Thus, the TCA cycle has a significance in the alleviation of ammonium toxicity.

Based on the results of RNA-seq, we found *P. notoginseng* roots in 15N and 15AN treatments have 117 and 268 common up- and down-regulated genes, respectively (Fig. 2c, d). Then the expression pattern of 5 and 3 significantly up- and down-regulated genes were confirmed using qRT-PCR, respectively (Fig. 4c to d, Additional file 1: Table S28, S29 and S32). These results show the expression of *ACLA-3* (XLOC-057761) is significantly up-regulated (Figure 6) in both 15N and 15AN treatments when compared to 15A treatment. This gene is homologous with *ACLA-3* (AT1G09430) of *A. thaliana*, mainly participate in modulating TCA cycle [44, 45]. Our results also indicated that several key metabolites, i.e., citrate, succinate, fumarate, and malate, in the TCA cycle are also significantly enhanced in both 15N and 15AN treatments (Fig. 6). These results suggest that nitrate addition can activate TCA cycle by up-regulating *ACLA-3* to synthesize more *α*-ketoglutarate for accelerating NH 4+ assimilation, and alleviate the ammonium toxicity (Fig. 6), which is also noticed in other species [20]. Thus, the activation of TCA cycle represents a conserved mechanism for the recovery of plant growth when adding nitrate to plants under ammonium stress.

As reported previously, ammonium toxicity causes an imbalance of carbon/nitrogen metabolism [39, 40]. Therefore, maintaining carbon/nitrogen metabolism balance can effectively avoid ammonium toxicity, which means to reduce the uptake of NH 4+ or to increase *α*-ketoglutarate content. Several researches indicated that addition NO 3− [10, 46] can reduce the uptake of NH 4+ to alleviate ammonium toxicity. Hachiya et al. [47] further supported the idea of NO 3− addition can up-regulated the expression of genes that related to nitrate signal in *A. thaliana*. Moreover, Yang et al. [15] showed ammonium and nitrate combination can increase auxin content to alleviate ammonium toxicity of *A. thaliana*; Zheng et al. [13] found NO 3− addition can regulate slow anion channels genes (*SLAH3*) to alleviate ammonium toxicity of *A. thaliana*. Our results show that addition of NO 3− can alleviate ammonium toxicity in *P. notoginseng* root potentially by activating *ACLA-3* and the TCA cycle, as reported in other species [20]. To summarize, the alleviation of ammonium toxicity when adding nitrate might be realized through multiple mechanisms.

An ABC transporter gene and a coiled-coil protein gene were also verified to have increased abundances in both 15N and 15AN treatments (Fig. 4a and c). ABC transporter is involves in the transportation of a large variety of nutrients, biosynthetic precursors, trace metals and vitamins, which is essential for the normal growth of plants [48–52]. Thus, the enhanced ABC transporter gene potentially contribute to the recovered growth when adding nitrates. The coiled-coil protein genes often involved in gene regulation whose relation to the relieved toxicity when adding nitrate will need more studies. Among the confirmed down-regulated genes in both 15N and 15AN treatments, DRM1/ARP (dormancy-associated gene-1/auxin-repressed protein) is a marker for dormancy release and expressed in tissues other than dormant axillary buds with high expression often localized to non-growing tissues [53]. Our results suggest that DRM1/ARP is repressed when adding nitrate to recover normal tissue growth (Fig. 4b and d). ACIK1 is serine/threonine-protein kinase At5g01020-like, whose relation to the relieved ammonium toxicity awaits further work. To summarize, these results suggest that there could be other parallel pathways that contribute to the alleviation of ammonium toxicity when adding nitrate (Table 1).

## Conclusion

Extravagant use of ammonium fertilization affected the quality of *P. notoginseng*, and reduced the overall root biomass of *P. notoginseng*. In this study, we found that ammonium stress leads to the destruction of plasma membrane integrity in roots of *P. notoginseng* and the inhibition of root growth. Using both RNA-seq and qRT-PCR, we identified that *ACLA-3* is up-regulated after introducing NO 3− to *P. notoginseng* roots under NH 4+ stresses, which contributes to the recovery of root integrity and growth. These results offer new insight toward a better understanding on the mechanism of higher yield of *P. notoginseng* root, as well as other crops, when ammonium and nitrate fertilizer are simultaneously used.

## Materials and methods

### Plant material

Xiaohong Ou and Xiuming Cui undertook the formal identification of the plant material used in the study. Two-year old *P. notoginseng* seedlings used for hydroponics were collected from the greenhouse of Faculty of Life Science and Technology, Kunming University of Science and Technology (24 ^∘^ 51 ^′^ 0 ^′′^ N, 102 ^∘^ 52 ^′^ 2 ^′′^ E, altitude 1835 m), Yunnan, China by Xiaohong Ou and Peiran Liao. Seedlings with similar growing status were randomly selected for experiments.

### Sandy culture

A pot with 10 kg quartz sand was used for a six months sandy culture. Four treatments with three replicates were selected, respectively were CK (Control); 15A (15 mM NH 4+); 15N (15 mM NO 3−); 15AN (15 mM NH 4++15 mM NO 3−). Hoagland’s solution was modified according to the N treatments, NO 3− was replaced with Ca(NO_3_)_2_, NH 4+ was replaced with (NH_4_)_2_SO_4_, NH 4++ NO 3− was replaced with NH_4_NO_3_. The final concentrations of the other macro-elements were 2.5 mM K_2_SO_4_, 7.5 mM CaSO_4_, 1 mM K_2_HPO_4_, and 1 mM MgSO_4_. The final concentrations of micro-elements were 50 *μ*M EDTA-Fe, 7 *μ*M Mn, 0.7 *μ*M Zn, 0.8 *μ*M Cu, 2 *μ*M B, 0.8 *μ*M Mo. To prevent the nitrification of NH 4+, 7 *μ*M dicyandiamide (a nitrification inhibitor) was added in all solutions. The solution pH was adjusted within the range of 6.0–6.1. Each pot was poured 1 L solution every week, and transplanted eight 2-yr old seedlings, others were same with regular managements. After six months cultivation, all the seedlings were harvested to determine plant height, leaf length and width, hair root length, rhizoma diameter, root diameter and biomass by Xiaohong Ou and Peiran Liao.

### Hydroponic culture

Xiaohong Ou and Peiran Liao used hydroponic culture to examine effects of different forms of nitrogen treatments on antioxidant system of *P. notoginseng* seedlings. Seedlings with similar growth vigor were washed off soil with tap water and washed three times with deionized water, then soaked in 2% NaClO for 2 h and washed up NaClO with deionized water. After that, seedlings were pretreating with completely nutrient solution for one week, and the solution was renewed every two days. The composition of the nutrient solution was referred to Roosta et al. [54], containing 0.25 mM NH_4_NO_3_, 0.2 mM KH_2_PO_4_, 0.2 mM K_2_SO_4_, 0.3 mM CaSO_4_, 0.3 mM MgSO_4_, 50 *μ*M EDTA-Fe, 7 *μ*M MnSO_4_, 0.7 *μ*M ZnCl_2_, 0.8 *μ*M CuSO_4_, 2 *μ*M H_3_BO_3_, 0.8 *μ*M Na_2_MoO_4_. The treatments and solutions composition of hydroponic culture were same with those of the sandy culture, which were CK (Control); 15A (15 mM NH 4+); 15N (15 mM NO 3−); and 15AN (15 mM NH 4+ + 15 mM NO 3−). Each treatment was replicated three times, and nutrient solution of each pot was renewed every two days. Six seedlings that pre-treated with above procedure were transplanted to each pot. On the third day, three seedlings of each treatment were harvested, then washed clean, divided into shoots and roots, frozen immediately with liquid nitrogen, stored at -80 ^∘^C for RNA extraction and metabolites determination. The hair roots of each seedling were used for root activity and plasma membrane integrity. In addition, the length of same hair root for each treatment was respectively measured on the first, third and fifth day.

### Examining root activity

Root activity was analyzed by the triphenyl tetrazolium chloride (TTC) method [55] by Xiaohong Ou and Peiran Liao. TTC is a chemical that is reduced by dehydrogenases, mainly succinate dehydrogenase, when added to a tissue. The dehydrogenase activity is regarded as an index of the root activity. Briefly, approximately 0.5 g fresh root was immersed in 10 mL of equally mixed solution of 0.4% TTC and phosphate buffer, and kept in the dark at 37 ^∘^C for 2 h. Subsequently, 2 mL of 1 mol/L H_2_SO_4_ was added to stop the reaction with the root. The root was dried with filter paper and then extracted with ethyl acetate. The red extractant was transferred into the volumetric flask to reach 10 mL by adding ethyl acetate. The absorbance of the extract at 485 nm was recorded. Root activity was expressed as TTC reduction intensity. Formally, root activity was calculated with the following equation, *a*=*r*/(*w*×*t*), where *a* is root activity (*μ*g/(g ×h)), *r* is TTC reduction (*μ*g), *w* is the fresh root weight (g), and *t* is time (h).

### Examining root plasma membrane integrity and relative root elongation

Hair roots on the third day of treatments were used to examine the root plasma membrane integrity and relative root elongation by Xiaohong Ou and Peiran Liao. Evans blue staining indicates cell death, and the degree of staining in the root provides a semi-quantitative measurement of membrane permeability. Briefly, approximate one cm of the root tips were used for plasma membrane integrity by using the Evans-Blue (E-B) staining method [56]. Hair roots were thoroughly rinsed with deionized water, gently blotted and weighed. *P. notoginseng* root tips were stained with 0.5% (v/v) E-B solution for 10 min at room temperature, rinsed three times with deionized water for a total of 10 min and photographed. Relative root elongation (%) is calculated by (root length increment of treatment / root length increment of control) ×100%.

### RNA extraction and sequencing

Briefly, total RNA was extracted from each root tissue using the TRIzol reagent (Invitrogen, Thermo Fisher Scientific Inc., USA) and digested with DNase I (Takara, Dalian, China) according to the manufacturer’s protocol. Then, the integrities of the RNAs were checked using an ultraviolet spectrophotometer (Hoefer, MA, USA), based on the ratio of the optical density at 260 nm to that at 280 nm (OD260/280 = 1.8—2.0). And total RNA were also assessed by electrophoresis in a agarose gel, which was visually judged the integrity of 18s and 28s ribosomal RNAs. After using an Agilent 2100 Bioanalyzer to determine the quality and concentration of each sample. The RNA-Seq libraries was constructed according to the manufacturer’s suggestion. The RNA-Seq libraries were sequenced by Illumina Hiseq 2000 sequences at BGI, shenzhen, China. The obtained RNA-Seq profiles had been deposited to NCBI under GEO the accession number, GSE112437. The qualities of the obtained RNA-Seq profiles were evaluated with the FASTQC program (https://www.bioinformatics.babraham.ac.uk/projects/fastqc/).

### Bioinformatics analysis of RNA-seq profiles

The raw reads of the datasets were aligned to the genome of *P. notoginseng* [21]. The reference genome [21] was built the index of using Bowtie2 v2.1.0 [57]. We used Tophat v2.0.13 [58] to align the sequencing reads to the reference genome [21] with the options of “-a 6 -r 50 -G”. Then, Cufflinks (v2.2.1) was used to assemble, Cuffmerge (v2.2.1) was used to merge the transcriptomes with the options of “- 40”, Cuffcompare (v2.2.1) was used to compared the self-assembled to the reported gene annotation with its default parameters [21], Cuffquant (v2.2.1) was used to calculate the gene expression levels with the options of “-u -p 4", and Cuffnorm (v2.2.1) was used to normalize the gene expression levels as FPKM with the options of “-p 16" (Fragments Per Kilo basepairs per Million sequencing tags) [22]. The assembled genes were annotated by being aligned to the NCBI non-redundant nucleotide (Nt) database with BLASTN and NCBI non-redundant nucleotide protein (Nr) database using BLASTX with the option of “-e 1E-5”.

Gene expression levels under one of the groups with different treatments greater than 5 were used to perform Principal Component Analysis (PCA) analysis and clustering analysis. FPKM values plus one of genes were log2-scaled and then applied to the prcomp function in the psych library in R to perform PCA. Then the log2-scaled FPKM values of genes were used to calculate the correlation coefficient matrix of samples which was applied to the pheatmap function in the pheatmap library in R to perform hierarchical clustering of samples. The abundances of genes were compared to control to identify Differentially Expressed Genes (DEGs) using the edgeR package [23]. Genes with a FDR <0.05 values and fold change |log_2_FC |>1 values were used as the significantly DEGs. We selected 117 up-regulated and 268 down-regulated genes under 15N and 15AN treatments but not under 15A treatment to perform a bi-clustering with the clustergram function in MatLab (MathWorks, Natick, MA, USA). KOBAS 3.0 was used to obtain Gene Ontology (GO) term and Kyoto Encyclopedia of Genes and Genomes (KEGG) pathway [59, 60]. The GO terms with multiple test corrected *P*-values ≤0.05 and KEGG pathways with *P*-values ≤0.05 were regarded as significantly enriched terms and pathways, respectively.

### Real-time PCR

We collected roots of *P. notoginseng* plants grown in Kunming, Yunnan, China to validate the expression levels using the quantitative real-time PCR (qRT-PCR) experiments. On the third day of the hydroponic culture, three seedlings of each treatments were collected, root of each seedling was separated and frozen by liquid nitrogen, then stored at -80 ^∘^C. Total RNA extraction of each sample was same with that for sequencing. cDNA synthesis was referred to Livak et al. [61]. Briefly, 4 *μ*g of total RNA was mixed with 1 *μ*l of Oligo (dT _18_) primer (Thermo, Thermo scientific technology, USA), 200 U of Revert Aid M-MuL Virus RT (Thermo) in the presence of 20 U of RiboLock RNase inhibitor (Thermo). Oligonucleotide primers for genes were designed using Primer Express Software (Applied Biosystems, Foster City, CA). After RT, 20 ng of cDNA from the same cDNA batch was subjected to RT-PCR to amplify all genes in triplicate in a total reaction volume of 15 *μ*l using Roche SYBR Green Master mix (Roche, Basel, Swiss), and the required amount of forward and reverse primers. Reactions were conducted on a LightCycler (Roche, Roche Life Science, CH) using the following cycling conditions: pre-incubation at 95 ^∘^C for 10 min, 3-step amplification at 95 ^∘^C for 10 sec, 60 ^∘^C for 30 sec, and 72 ^∘^C for 1 min. All samples were run in triplicate and each cDNA sample was run in duplicate. Expression of *P. notoginseng* *ACT*2 was used as an internal control for target gene expression. The gene expression was calculated based on the 2 ^−△△*Ct*^ method [61]. All the primer sequences of DEGs for real-time PCR were designed by Primer 5.0 software (Additional file 1: Table S28), and primers were synthesized by Tsingke Genomics Institute (Kunming, China).

### Assay method

#### NH 4+ determination

NH 4+ concentration was determined according to Husted et al. [62]. 0.5 g fresh root tissue was homogenized with 5 ml deionized water on ice, then centrifuged at 25,000 g (2 ^∘^C) for 10 min. The supernatant was used for NH 4+ concentration determination by colorimetric.

#### Amino acids determination

Amino acids were measured by HPLC according to Geiger et al. [63]. Approximately 60 mg root was placed in a screw cap tube and extracted sequentially for 15 min at 70] ^∘^C with two 250 *μ*l of 80% aqueous ethanol (buffered with 10 mM HEPES-KOH, pH 7.0), one 250 *μ*l of 50% aqueous ethanol (buffered with 10 mM HEPES-KOH, pH 7.0) and one 250 *μ*l of 10 mM HEPES-KOH, pH 7.0. The extract was centrifuged between each step (5 min, 14,000 g). The supernatants were combined and measured by HPLC (Agilent 1120, Palo Alto, CA, USA).

#### Carbohydrate determination

Total soluble sugar concentration was determined by anthrone method according to Siddiqi et al. [64]. 0.5 g root sample was homogenized using a mortar and pestle with 10 ml distilled water, then centrifuged at 1,000 ×g for 10 min, the supernatant was collected. After appropriate dilution of the supernatant, soluble sugar was colorimetric assayed at 630 nm by adding concentrated HCl, 45% formic acid, and anthrone/sulphuric acid solution.

Glucose and sucrose were measured by spectrophotometry method according to Stitt [65]. 0.5 g root sample was homogenized with 10 ml 80% ethanol, then centrifuged at 3,000 ×g for 10 min. The supernatant was used for glucose and sucrose measurement.

#### Organic acids determination

0.5 g fresh root sample homogenized with 1.5 ml 5% trichloroacetic acid, centrifuged at 10,000 ×g for 10 min, supernatant was used for organic acids determination [25]. Organic acids analyzed by HPLC method with Aglient 1200 (USA) and an Intramax (4.6 mm ·250 mm, 5 *μ*m) column. Mobil phrase was 3% methanol 0.01 M K_2_HPO_4_ (pH=2.55), flow rate was 0.5 ml ·min^−1^, column temperature was 30 ^∘^C, injection volume was 10 *μ*l, detective wavelength was 210 nm [66].

#### Enzymatic assays

Enzymatic assays were determined according to Sarasketa et al. [11]. Fresh roots were homogenized using a mortar and pestle with 20 *μ*l of extraction buffer per mg of FW [10 mM MgCl_2_, 1 mM EDTA, 1 mM EGTA, 10 mM dithiothreitol (DTT), 0.1% Triton X-100, 10% glycerol, 0.05% bovine serum albumin (BSA), 0.5% polyvinylpolypyrrolidone (PVPP), 50 mM HEPES pH 7.5] in the presence of a cocktail of proteases inhibitors [1 mM phenylmethylsulfonyl fluoride (PMSF), 1 mM *ε*-aminocaproic acid, 10 *μ*M leupeptin]. Homogenates were then centrifuged at 4,000 g for 30 min at 4 ^∘^C and the supernatants recovered.

For phosphoenolpyruvate carboxylase (PEPC): 100 mM Tricine-KOH (pH=8), 5 mM MgCl_2_, 5 mM NaF, 0.25 mM NADH, 6.4 U of malate dehydrogenase/ml, 2 mM NaHCO_3_ and 3 mM phosphoenolpyruvate; for MDH: 100 mM HEPES-KOH (pH=7.5), 5 mM MgSO_4_, 0.2 mM NADH, 2 mM oxaloacetate; for NADP-dependent isocitrate dehydrogenase (ICDH): 100 mM Tricine-KOH (pH=8), 0.25 mM NADP, 5 mM MgCl_2_, and 5 mM isocitrate.

#### Statistical analysis

All the data analyses were conducted with SPSS 18.0 (USA), Student’s *t*-test was used to compare the significant difference between the data with CK or ammonium treating and that with nitrate or combination treating.

## Supplementary information

**Additional file 1** Supplementary tables. This is an MS Excel file. This file includes 32 supplementary tables.

**Additional file 2** Supplementary figures. This is a pdf file. This file includes 7 supplementary figures.

## Data Availability

The datasets generated during and/or analyzed during the current study are available in the NCBI GEO database, https://www.ncbi.nlm.nih.gov/geo/query/acc.cgi?acc=GSE112437.

## Abbreviations

*ACLA-3*: ATP-citrate lyase A-3
BSA: Bovine serum albumin
CK: Control
DEGs: Differentially expressed genes
DTT: Dithiothreitol
E-B: Evans-Blue
FDR: False discovery rate
FPKM: Fragments per kilo basepairs per million sequencing tags
GO: Gene ontology
ICDH: NADP-dependent isocitrate dehydrogenase
KEGG: Kyoto encyclopedia of genes and genomes
*P. notoginseng*: *Panax notoginseng*
PCA: Principal component analysis
PEPC: Phosphoenolpyruvate carboxylase
PMSF: Phenylmethylsulfonyl fluoride
PVPP: Polyvinylpolypyrrolidone
TTC: Triphenyl tetrazolium chloride
