# Supplementary material for: The transcriptome variations of Panaxnotoginseng roots treated with different forms of nitrogen fertilizers
Source: BMC Genomics. 2019 Dec 24;20(Suppl 9):965. doi: 10.1186/s12864-019-6340-7 (PMC6929466; doi:10.1186/s12864-019-6340-7)
Supplement: Supplementary file 2 — Additional file 2 Supplementary figures. This is a pdf file. This file includes 7 supplementary figures. [file 12864_2019_6340_MOESM2_ESM.pdf]

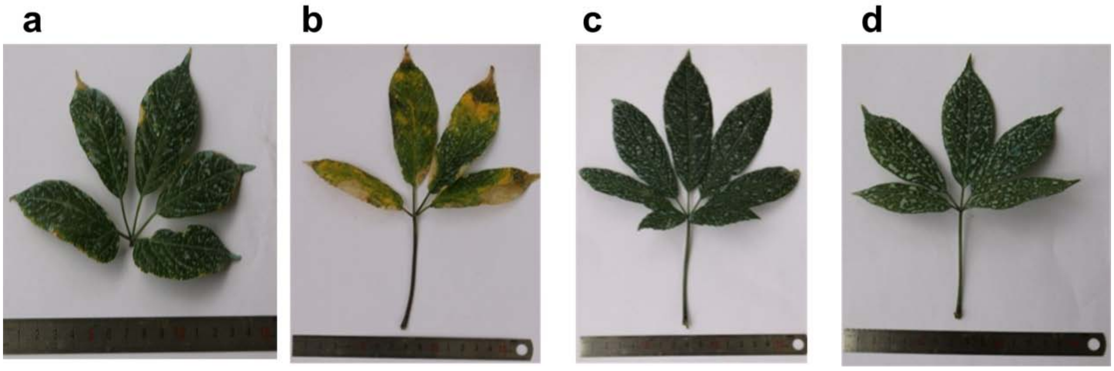

Figure S1. Effects of different forms of nitrogen treatments on the growth of *P. notoginseng* plants. The plant growth conditions was observed at 6 months after transplant. (a) Control. (b) NH_4^+ treatment. (c) NO_3^- treatment. (d) NH_4^+ and NO_3^- mixed processing.

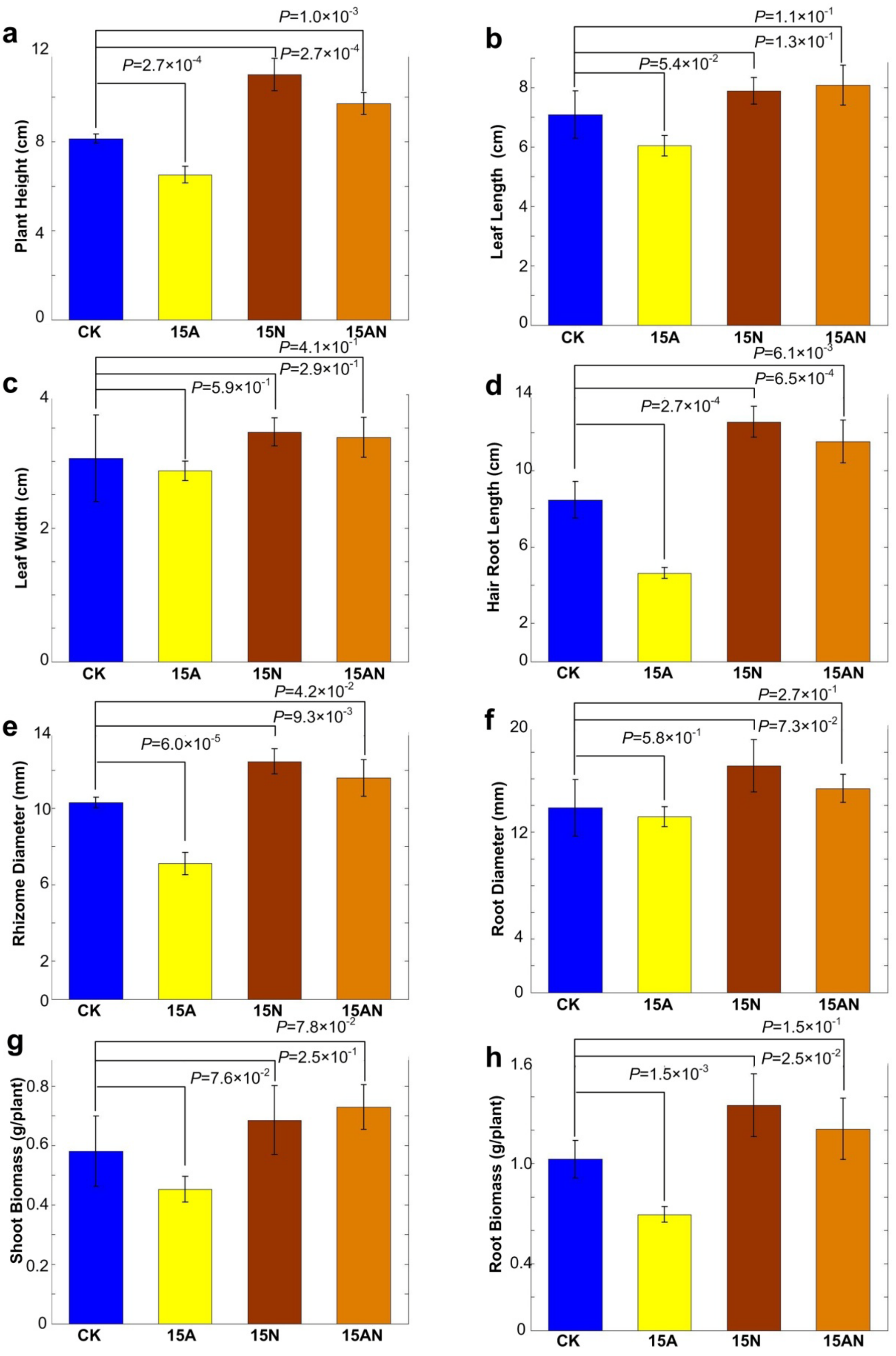

Figure S2. The plant growth status of *P. notoginseng* in sandy culture experiments. The horizontal axis is the different forms of nitrogen treatments. The names of different treatments are CK (Control); 15A (15 mM NH_4^+); 15N (15 mM NO_3^-); and 15AN (15 mM NH_4^++15 mM NO_3^-). The vertical is the values of different measures. The values shown are mean values, and error bars stand for standard deviations. The

P-values indicate significant differences compared with the CK treatment under the same treatment conditions (Student's *t*-test). The asterisks indicate significant differences compared with the 15A treatment under the same treatment conditions (Student's *t*-test). Standard deviations were calculated from three technical replicates. The results shown were reproduced with three biological replicates. (a) Plant Height. (b) Leaf Length. (c) Leaf Width. (d) Hair Root Length. (e) Rhizome Diameter. Rhizome is the underground part of the stem. (f) Root Diameter. (g) Shoot Biomass. (h) Root Biomass.

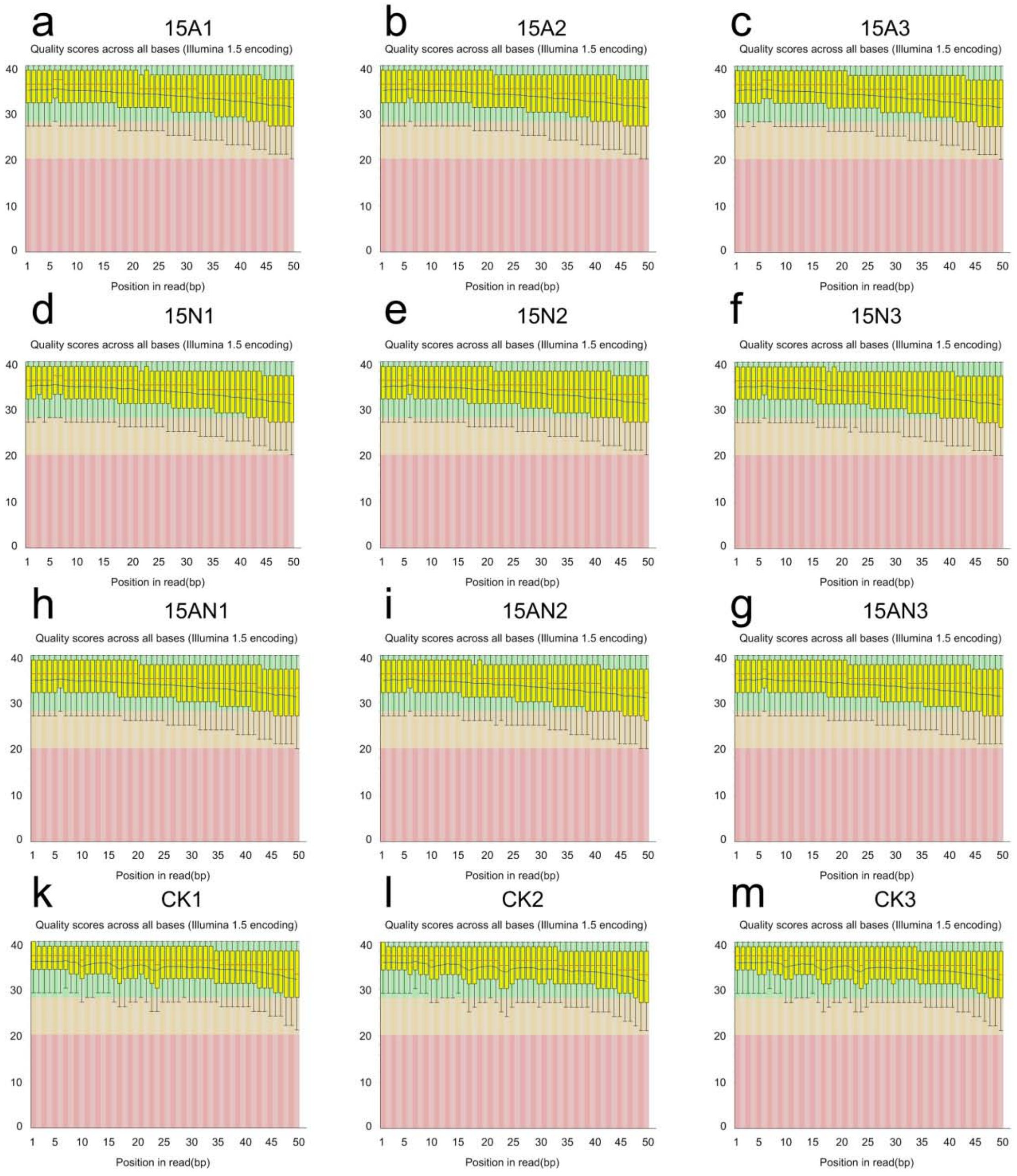

Figure S3. Per base sequencing quality of 12 RNA-seq sequencing profiles. The horizontal axis is the position of the reads. The vertical is the quality scores of the reads. (a) to (c): the values for the three samples under15 mM NH_4^+ treatment. (d) to (f): the values for the three samples under 15 mM NO_3^- treatment. (h) to (g): the values for the three samples under 15 mM NH_4^+ and NO_3^- mixed processing. (k) to (m): the values for the three samples in control treatment.

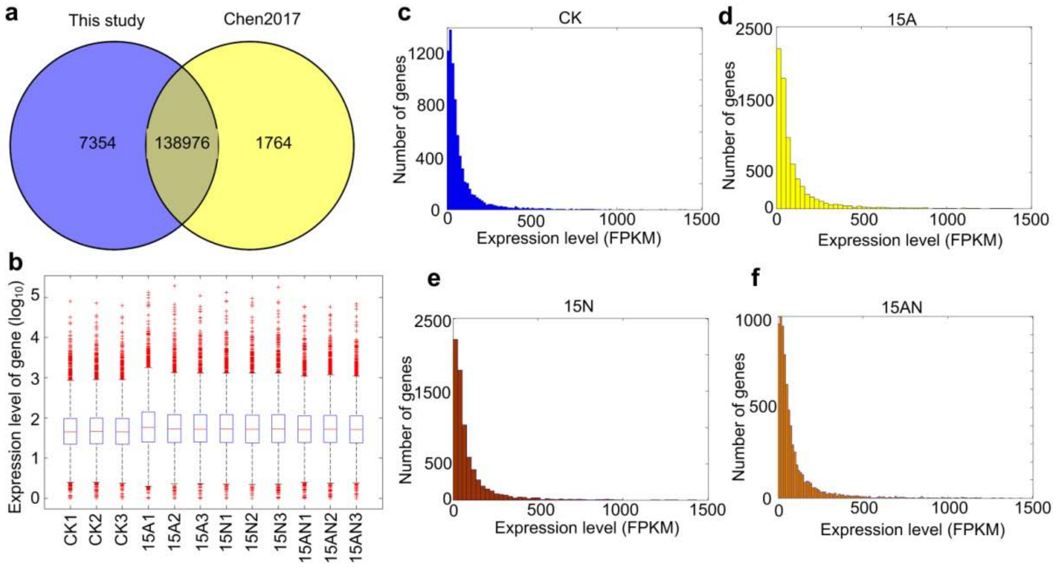

Figure S4. General characteristics of the self-assembled transcriptome of *P. notoginseng*. (a) The numbers of genes identified in our assembly and those reported in the previous study (Chen et al. 2017). (b) Average gene expression levels (in $\log_{10}$) in the samples of four different treatments. (c) to (f), histograms of genes versus average expression levels in the four groups of different treatments.

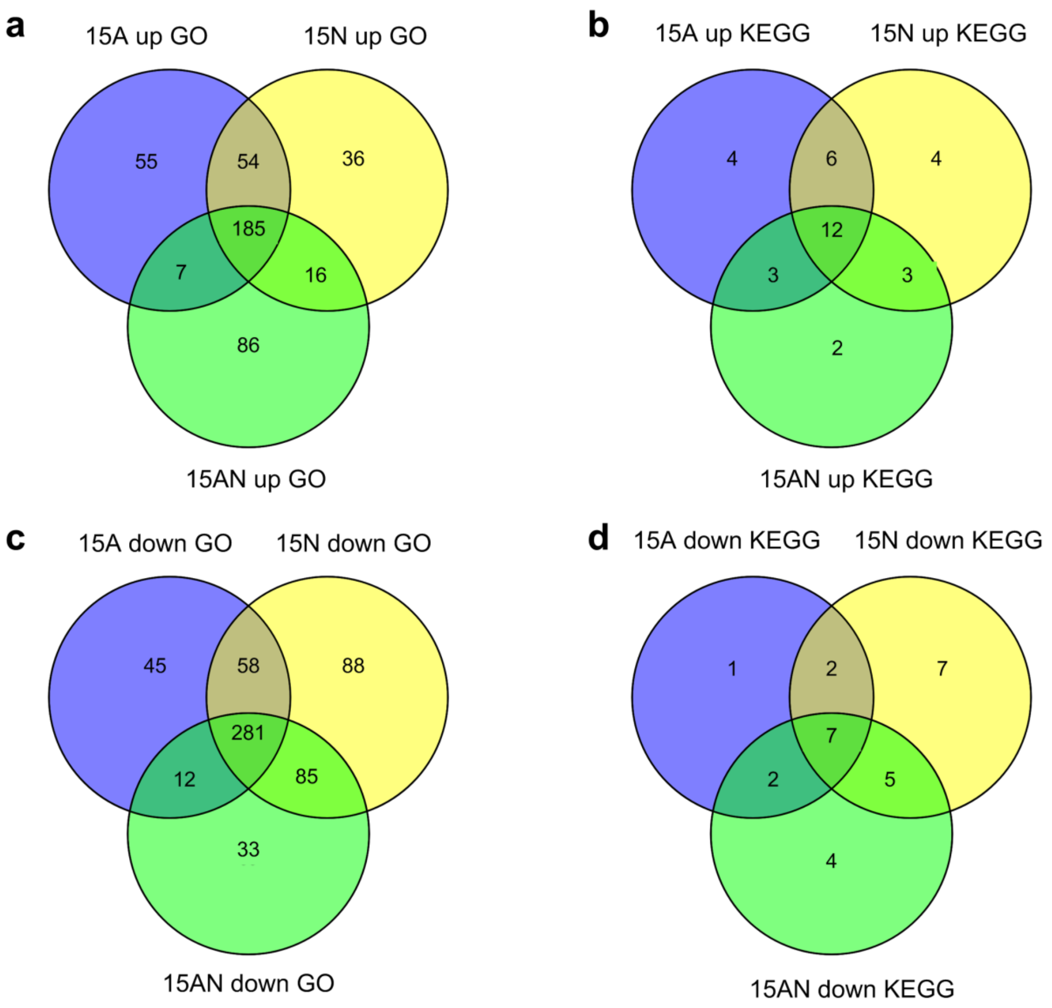

Figure S5. The numbers of significantly enriched GO terms and KEGG pathways for up-regulated and down-regulated genes in 15A (15 mM NH_4^+), 15N (15 mM NO_3^-) and 15AN (15 mM NH_4^+ and 15 mM NO_3^-) treatments. The GO terms with multiple test corrected *P*-values < 0.05 and KEGG pathways with *P*-values < 0.05 were regarded as significantly enriched terms and pathways, respectively. (a) to (b), the numbers of GO terms and KEGG pathways enriched in up-regulated genes, respectively. (c) to (d), the numbers of GO terms and KEGG pathways enriched in down-regulated genes.

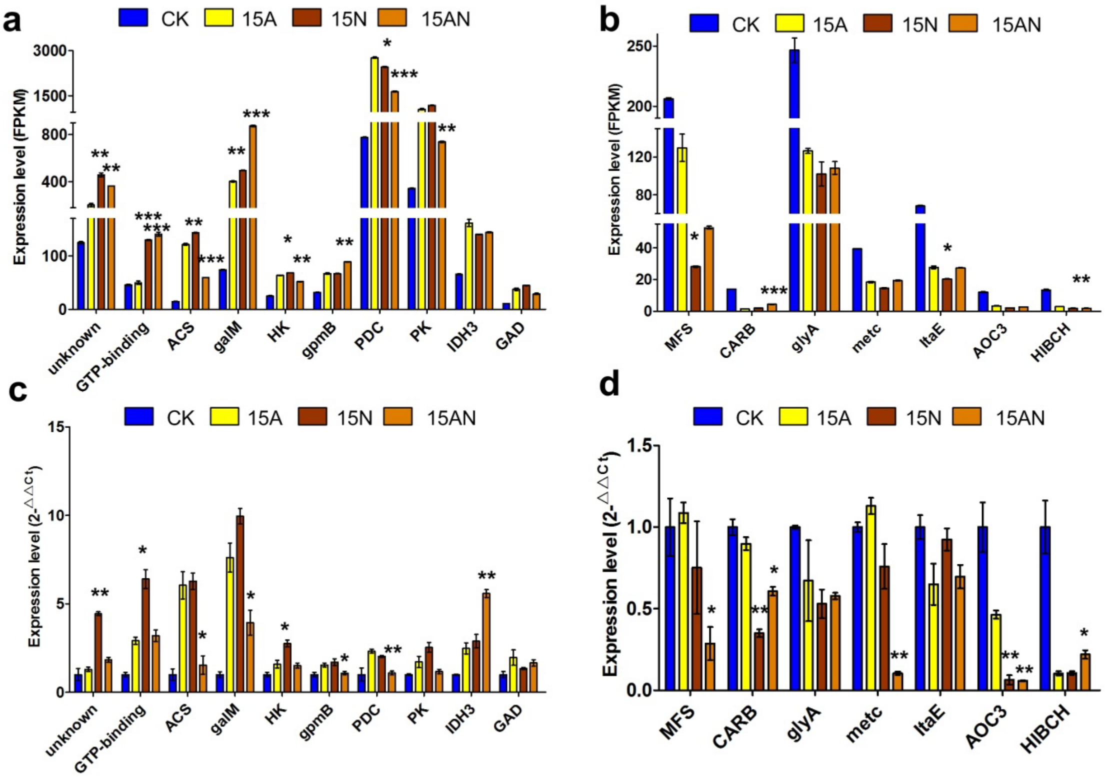

Figure S6. Validating expression patterns of selected up- or down-regulated genes by qRT-PCR in *P. notoginseng*. The asterisks indicate significant differences compared with the 15A treatment under the same treatment conditions (Student's *t*-test). Standard deviations were calculated from three technical replicates. The results shown were reproduced with three biological replicates. (a) to (b), the abundances (FPKM) of up-regulated and down-regulated genes obtained in the RNA-seq profiles, respectively. (c) to (d), the abundances ($2^{-\Delta\Delta Ct}$) of up-regulated and down-regulated genes obtained in the qRT-PCR experiments, respectively.

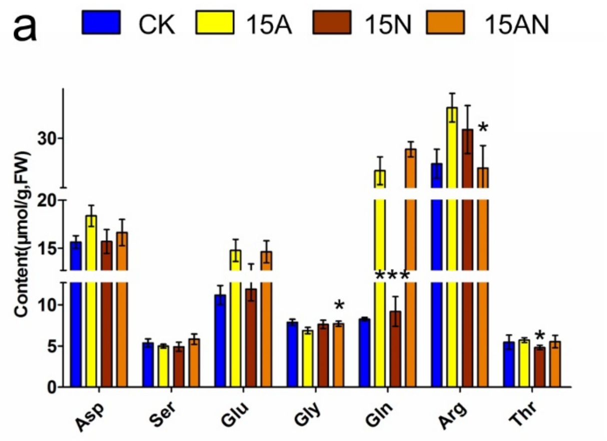

Figure S7. Effects of the different forms of nitrogen treatments on changes of the main amino acid contents of *P. notoginseng*. The asterisks indicate significant differences compared with the 15A treatment under the same treatment conditions (Student's *t*-test). Standard deviations were calculated from three technical replicates. The results shown were reproduced with three biological replicates.
